# Synthesis of (*E*)-2-(1*H*-tetrazole-5-yl)-3-phenylacrylenenitrile derivatives catalyzed by new ZnO nanoparticles embedded in a thermally stable magnetic periodic mesoporous organosilica under green conditions

**DOI:** 10.1038/s41598-022-13011-9

**Published:** 2022-06-24

**Authors:** Sajedeh Safapoor, Mohammad G. Dekamin, Arezoo Akbari, M. Reza Naimi-Jamal

**Affiliations:** Pharmaceutical and Heterocyclic Compounds Research Laboratory, Department of Chemistry, Iran University of Science and Technology, Tehran, 16846-13114 Iran

**Keywords:** Catalysis, Coordination chemistry, Environmental chemistry, Green chemistry, Inorganic chemistry, Materials chemistry, Medicinal chemistry, Organic chemistry, Surface chemistry, Chemical synthesis

## Abstract

ZnO nanoparticles embedded in a magnetic isocyanurate-based periodic mesoporous organosilica (Fe_3_O_4_@PMO–ICS–ZnO) were prepared through a modified environmentally-benign procedure for the first time and properly characterized by appropriate spectroscopic and analytical methods or techniques used for mesoporous materials. The new thermally stable Fe_3_O_4_@PMO–ICS–ZnO nanomaterial with proper active sites and surface area as well as uniform particle size was investigated for the synthesis of medicinally important tetrazole derivatives through cascade condensation and concerted 1,3-cycloaddition reactions as a representative of the Click Chemistry concept. The desired 5-substituted-1*H*-tetrazole derivatives were smoothly prepared in high to quantitative yields and good purity in EtOH under reflux conditions. Low catalyst loading, short reaction time and the use of green solvents such as EtOH and water instead of carcinogenic DMF as well as easy separation and recyclability of the catalyst for at least five consecutive runs without significant loss of its activity are notable advantages of this new protocol compared to other recent introduced procedures.

## Introduction

Since it’s first introduction by K. Barry Sharpless in 1999, “Click Chemistry (CC)” has been emerged as a very popular topic for the synthesis of heterocyclic compounds. In 2001, Sharpless defined CC reactions as a set of organic transformations with specific characteristics such as modular, wide in scope, affording high yields and produce only harmless byproducts that can be removed by non-chromatographic separation techniques^1–7^. 1,3-dipolar cycloaddition reactions are one of the most popular CC reactions^8^. When such concerted reactions are performed through multicomponent reaction (MCR) strategy, they can be widely used for the synthesis of important heterocyclic compounds including triazole and tetrazole derivatives^9–15^.

**Figure 1:**
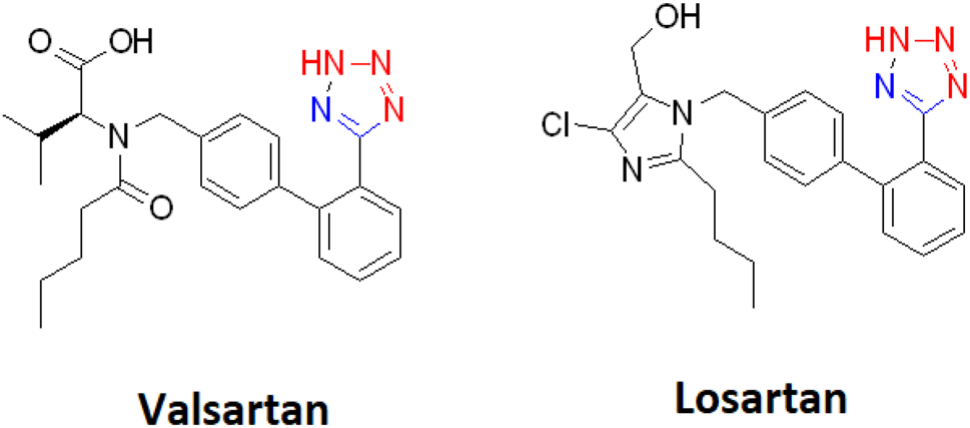
Valsartan and losartan as two active pharmaceutical ingredients with tetrazole moiety.

The presence of four nitrogen atoms in the heteroaromatic five-membered ring of tetrazole gives rise to nitrogen-rich planar structural features^16–21^. Furthermore, the acidic nature of tetrazoles is due to the presence of free N–H in their structure and this property can lead to the formation of more complex aliphatic and aromatic heterocyclic compounds through nucleophilic substitution^22,23^. Indeed, the heterocyclic tetrazole moiety can stabilize the negative charge of the corresponding anion by charge displacement and show same p*K*a values of the corresponding carboxylic acids^24^. As a result, tetrazoles can be used as a metabolic substitutes (bioisoesteres) for the carboxylate functional group. Hence, these two groups of organic compounds are similar at p*K*a = 4.9 and become deprotonated at physiological pH^25^. Also, tetrazoles have higher nitrogen content than other heterocycles and require almost the same electronic space as carboxylates. Consequently, these features have improved their uses in a wide range of applications including pharmaceuticals and drug design, food industries, explosives, agrochemicals, materials science, coordination chemistry, etc.^26–36^. Especially, the tetrazole structures are similar to the pharmacological core of the Saran’s family, which are in fact angiotensin II receptor blockers (ARB). Angiotensin II is a bioactive peptide that narrows the vessels through the contraction of the muscles around the heart^37^. These drugs are used in to lower blood pressure and heart failure. Among the most important pharmaceuticals in this category of drugs are losartan and valsartan (Fig. 1). Indeed, these two active pharmaceutical ingredients are the first of a new class of drugs, which have been introduced for the clinical use in hypertension^38,39^. Furthermore, many chemical studies on other tetrazole analogue compounds have described antibacterial and antifungal properties. Tetrazole derivatives also show anti-inflammatory, analgesic, anti-cancer, anticonvulsant and antidiabetic kidney disease activities^32^. Therefore, tetrazole chemistry has yet remained as a fascinating research area for different scientists.

Since the preparation of the first tetrazole compound in more than a hundred years ago, many scientists have invented or described different methods for preparation of tetrazole compounds^35^. The most common of these methods is a 1,3-dipolar cycloaddition reaction between different simple nitrile derivatives and azide ion or hydrazoic acid under pressure. This approach has been more developed using different catalytic systems or altering of substrates during different decades^35,40–42^. Some recent examples include the use of L-cysteine complex of palladium onto mesoporous channels of MCM-41^43^, Fe_3_O_4_@L-lysine-Pd(0)^43^, copper(I) salt^26^, copper catalyst on biochar nanoparticles^44^, guanidine complex of copper supported on boehmite nanoparticles^45^, zinc(II) salts^46^, Zn/Al hydrotalcite^47^, NiFe_2_O_4_^48^, TMSCl and Bu_2_SnO or *N*-methyl-2-pyrrolidone^49^, FeCl_3_–SiO_2_^50^, Amberlyst-15^28^, MCM-41-SO_3_H^51^, 1-disulfo-[2,2-bipyridine]-1,1-diium chloride ionic liquid^52^ mainly at elevated temperatures, or dialkyl aluminum azides as the substrate and catalyst^36^. Furthermore, Sharples and his colleague have reported improved intermolecular [3 + 2] ring closures between an azide and *p*-toluenesulfonyl cyanide or acyl cyanide under solvent-free conditions at elevated temperatures to afford good to excellent yields of the corresponding products using easy procedurs^53,54^. Moreover, the use of more complex nitrile derivatives such as benzylidenemalononitriles would be more desirable in terms of abundance of functional groups in the corresponding products^55^. Indeed, this route for the synthesis of tetrazoles involves the multicomponent reactions (MCRs) strategy between aldehydes, sodium azide and nitriles. One of the advantages of this strategy is the use of available, easy and inexpensive basic compounds, namely aldehydes and sodium azide along with relatively expensive nitriles, which has made these reactions economically viable and easy to do on an industrial scale^56–60^. Among different catalytic systems for benzylidenemalononitrile derivatives, which are generally prepared by condensation of aldehydes and molononitrile, Fe_3_O_4_@BNPs-CPTMS-Chitosan-Pd(0)^61^, Cu(II) immobilized on Fe_3_O_4_@HNTs–tetrazole nanocomposite^62^, secondary amine/Cu(II) bifunctional magnetic nanoparticles^63^, Fe_3_O_4_ magnetic nanoparticles under microwave irradiation^64^, NiO_2_ nanoparticles^65^, silica molybdic acid^66^ and Cu_2_(BDC)_2_(DABCO) metal–organic framework^67^ could be mentioned. In spite of their merits, there are some disadvantages to the most of previously reported protocols for the synthesis of tetrazoles. These include low boiling point of hydrazoic acid (37 °C), the use of expensive catalytic systems, high pressure conditions, toxic and carcinogenic solvents including DMF, or elevated temperature for the explosive azide ion component.

Periodic mesoporous organosilicas (PMOs) have been also emerged as one of the important issues of research in recent years. PMOs which were reported for the first time in 1999 are a new branch of mesoporous materials. They are organic–inorganic hybrid materials with high-ordered structures and uniform pore sizes^68–82^. PMOs are essentially unique because of the advantage of combining a strong porous inorganic framework with the inherent properties of organic components having different functionalities^71,83–86^. In this regard, precursors of bridged organosilica bearing hetero-aromatic isocyanurate moieties with high thermal stability and low toxicity would be very desirable^82,85–88^. On the other hand, PMOs demonstrate other distinguishing characteristics such as large and hollow spaces, high surface area, regular cavity wall structure, low density and good membrane permeability, and material loading in large quantities. Therefore, PMOs have been effectively used in many applications such as drug and gene delivery, gas and molecule absorption, sensors, active agents for smart anticorrosive organic coatings, and catalysis^85,86,89–107^.

**Figure 2:**
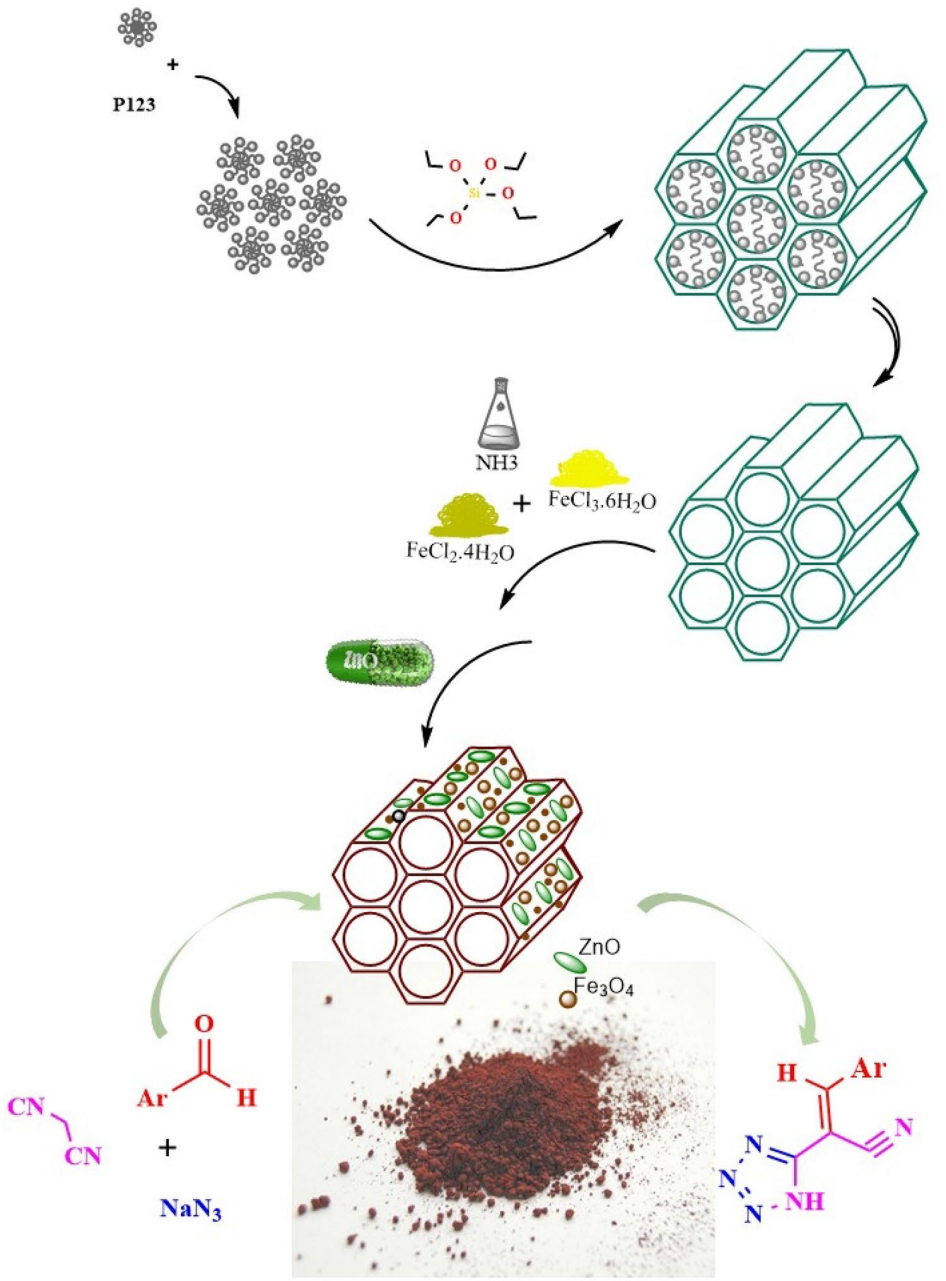
Schematic preparation of the Fe_3_O_4_@PMO-ICS–ZnO (**1**) catalyst and its application in the synthesis of tetrazoles derivatives **5a–o**.

To address the above challenges and in continuation of our studies to explore the catalytic activity of ZnO species^108,109^ as well as PMOs^91,94–99^, we wish to report herein the new ZnO nanoparticles embedded in a magnetic isocyanurate-based periodic mesoporous organosilica (Fe_3_O_4_@PMO–ICS–ZnO) for the cascade reaction of different aromatic aldehydes and malononitrile to afford the corresponding Knoevenagel intermediate, and subsequent [3 + 2] cycloaddition with sodium azide (Fig. 2). Indeed, diverse tetrazole derivatives **5a–o** were prepared efficiently using low loading of Fe_3_O_4_@PMO–ICS–ZnO, as a magnetically recoverable catalytic system, in EtOH under reflux conditions in short reaction times.

## Results and discussion

After preparation of the Fe_3_O_4_@PMO-ICS-ZnO nanomaterial, its structural, morphological and textural properties were characterized by Fourier transform infra-red (FTIR) and X-ray powder diffraction (XRD) spectroscopy, field emission scanning electron (FESEM) microscopy, thermogravimetric analysis (TGA), N_2_ adsorption–desorption isotherms and vibrating sample magnetometer (VSM).

**Figure 3:**
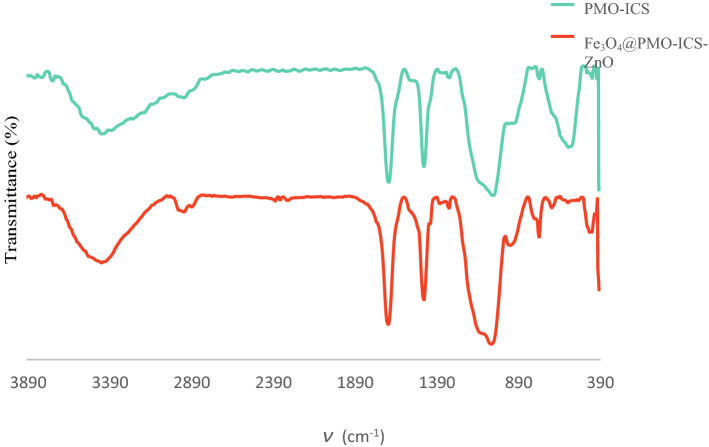
FTIR spectra of the PMO-ICS and magnetic Fe_3_O_4_@PMO-ICS-ZnO nanoporous catalyst (**1**).

The FT-IR spectra of both PMO-ICS and Fe_3_O_4_@PMO-ICS-ZnO have been presented in Fig. 3. It was observed that the free O–H stretching mode of the silanol groups on the PMO surface appear at 3415 cm^−1^. Also, sharp absorption bands were observed at 1689 and 1471, which are related to the vibrations of carbonyl and C–N bonds in the isocyanurate ring within the framework of the prepared nanocatalyst, respectively. The signals appearing in 2939 and 2889 cm^−1^ are related to the stretching vibrations of aliphatic moiety of PMO-ICS. Furthermore, the observed bands at 1108, 1056 and 943 cm^−1^ correspond to the asymmetric and symmetric stretching vibrations of siloxane Si–O–Si bonds. On the other hand, the signals at 570 cm^−1^ are related to the stretching vibration of the Fe–O–Fe bonds. The Zn–O stretching vibration modes are observed, as a relatively weak band at 493 cm^−1^, which merge with the nearby signal of Fe–O bonds.

**Figure 4:**
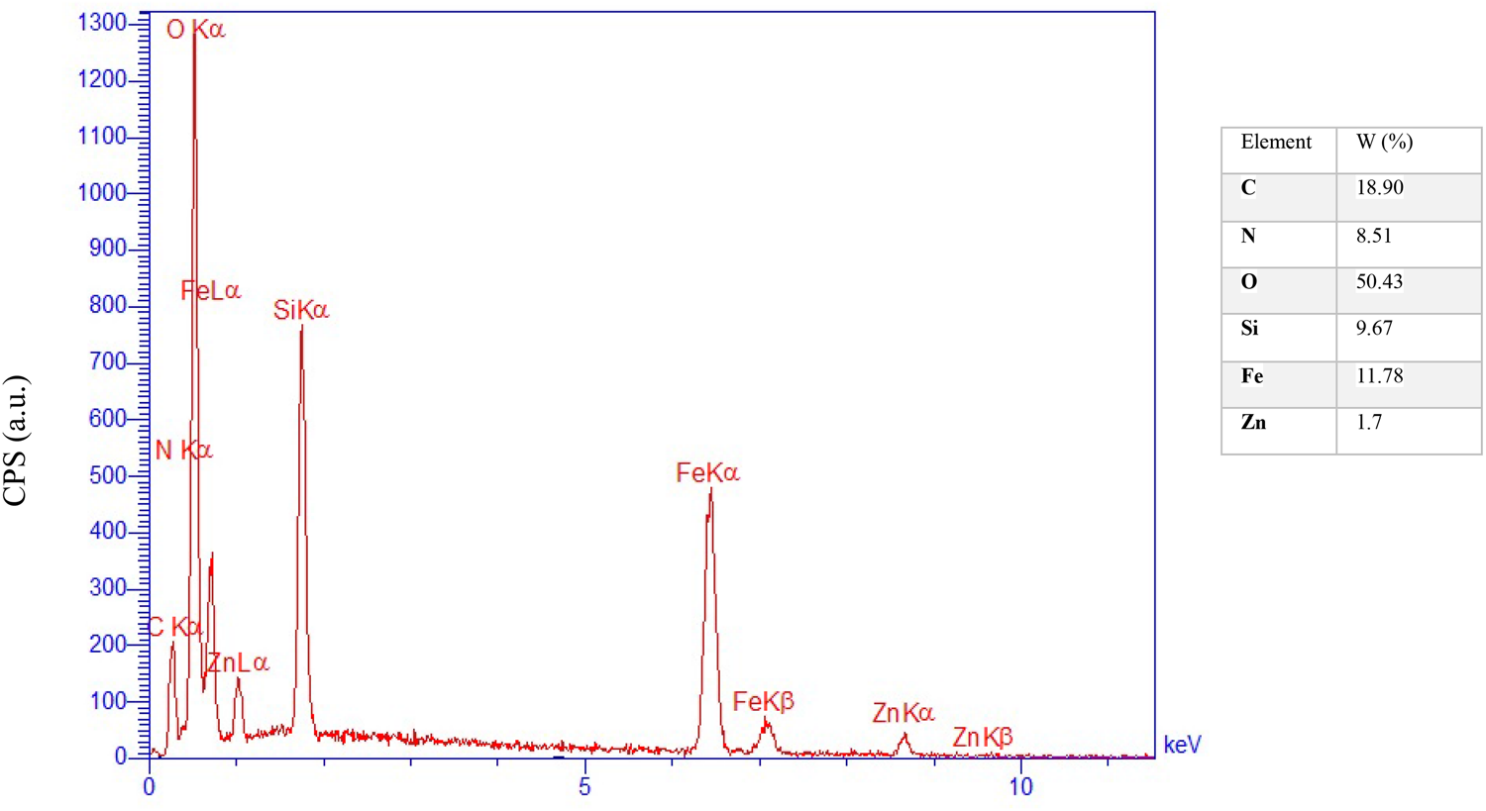
EDX spectrum of the magnetic Fe_3_O_4_@PMO-ICS-ZnO mesoporous catalyst (**1**).

The energy dispersive X-ray (EDX) spectrum of the catalyst has been shown in Fig. 4. Indeed, the signals of iron, nitrogen, silicon, carbon, oxygen and zinc atoms were observed in the studied sample. It is well known that three peaks of iron are observed in the EDX spectrum for magnetic iron oxides. Furthermore, this analysis successfully confirms that ZnO nanoparticles are well embedded into the magnetic catalyst.

**Figure 5:**
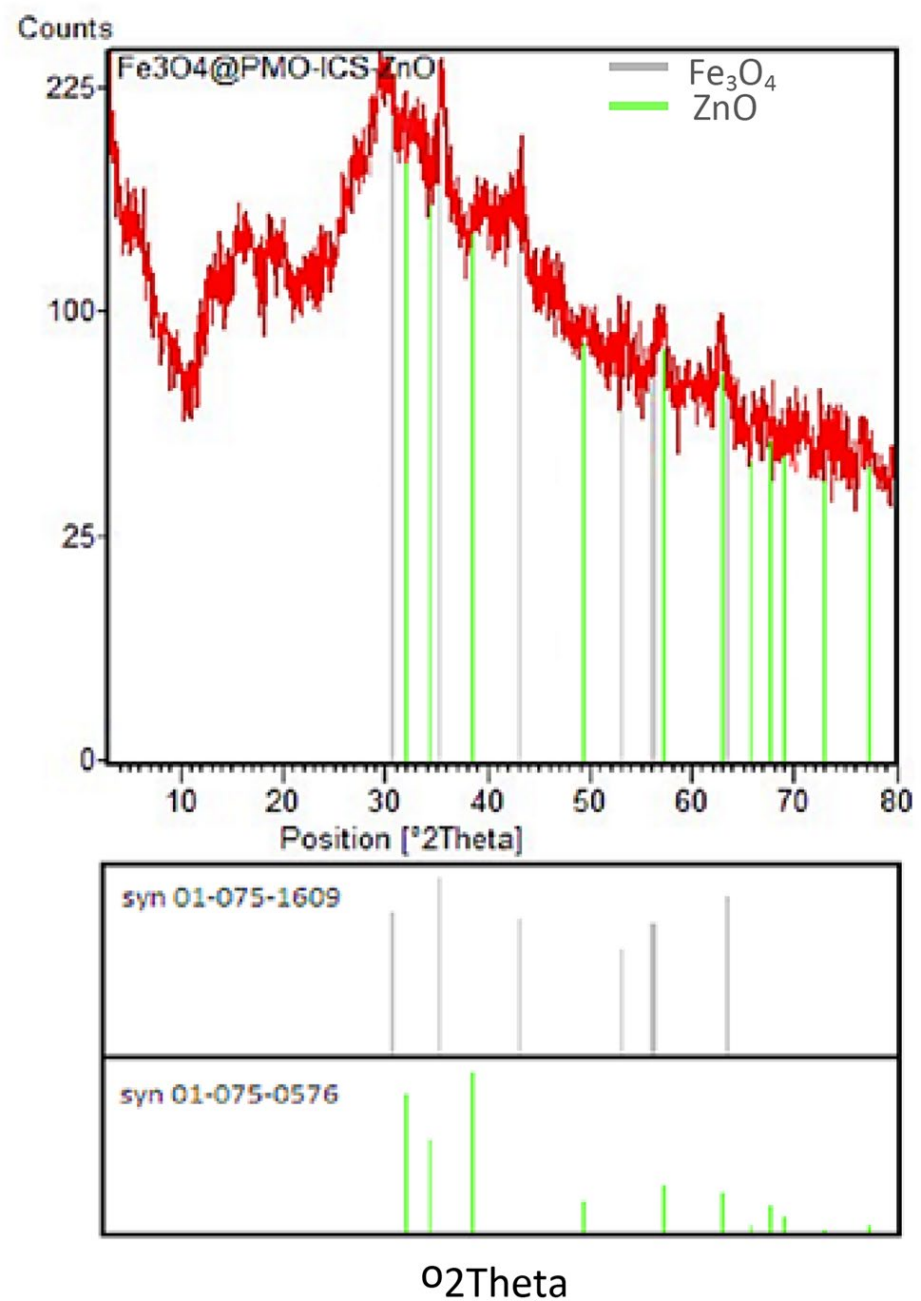
X-ray powder diffraction (XRD) pattern for the Fe_3_O_4_@PMO-ICS-ZnO nanoporous catalyst (**1**).

The structure of Fe_3_O_4_@PMO-ICS-ZnO nanoparticles was also analyzed by XRD spectroscopy. The wide angle XRD pattern shown in Fig. 5 determined the crystallinity and arrangement of both PMO and Fe_3_O_4_ components in the structure of Fe_3_O_4_@PMO-ICS-ZnO nanomaterial. Indeed, a broad diffraction peak of 2θ near to 3.9° and five sharp peaks at 2θ = 29.5°, 35.4°, 43.2°, 56.9°, and 62.7° demonstrate corresponding reflections of amorphous silica as well as Fe_3_O_4_ and ZnO phases, respectively.

**Figure 6:**
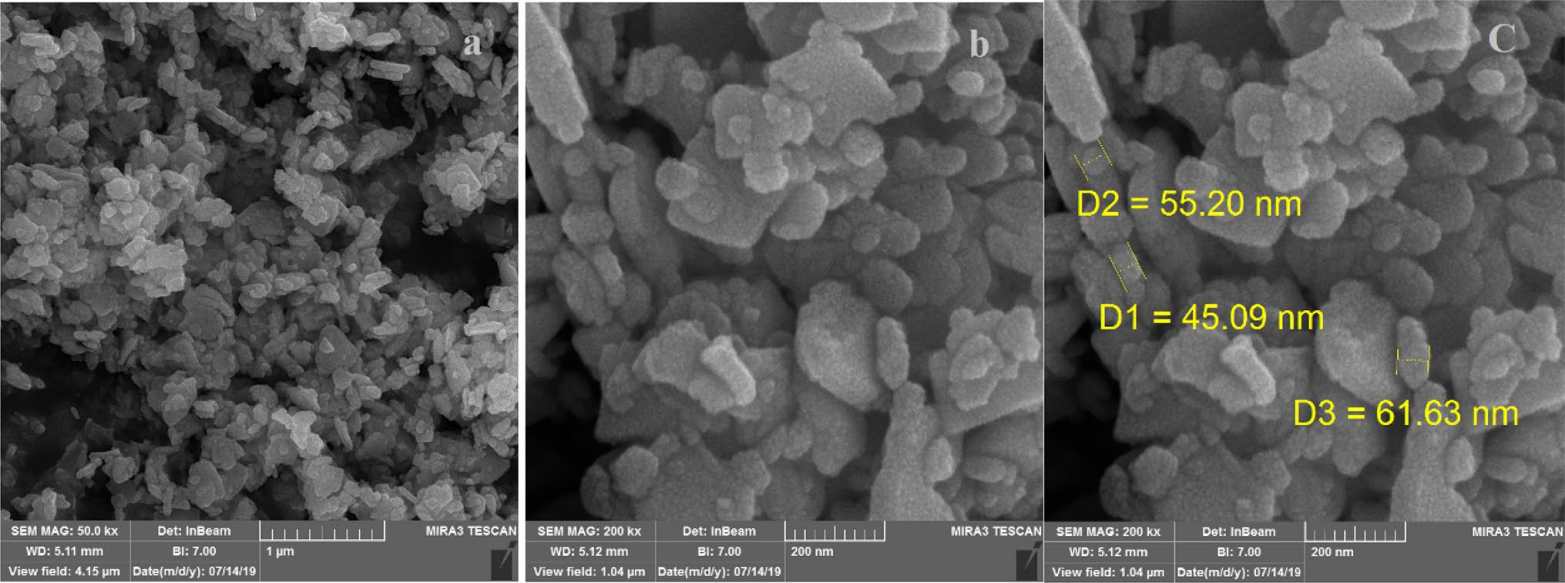
FESEM images of the magnetic nanoporous Fe_3_O_4_@PMO-ICS-ZnO catalyst (**1**).

The morphology, distribution and size of the particles of Fe_3_O_4_@PMO-ICS-ZnO nanoporous material (**1**) were analyzed using field emission scanning electron microscope (FESEM, Fig. 6). A uniform and well-defined distribution as well as almost nano-rod morphology were observed for the ZnO nanoparticles embedded in the magnetic periodic mesoporous organosilica. Furthermore, the average particle sizes of Fe_3_O_4_@PMO-ICS-ZnO nanoporous material (**1**) was found to be in the range of nano scale and about 45–62 nm.

**Figure 7:**
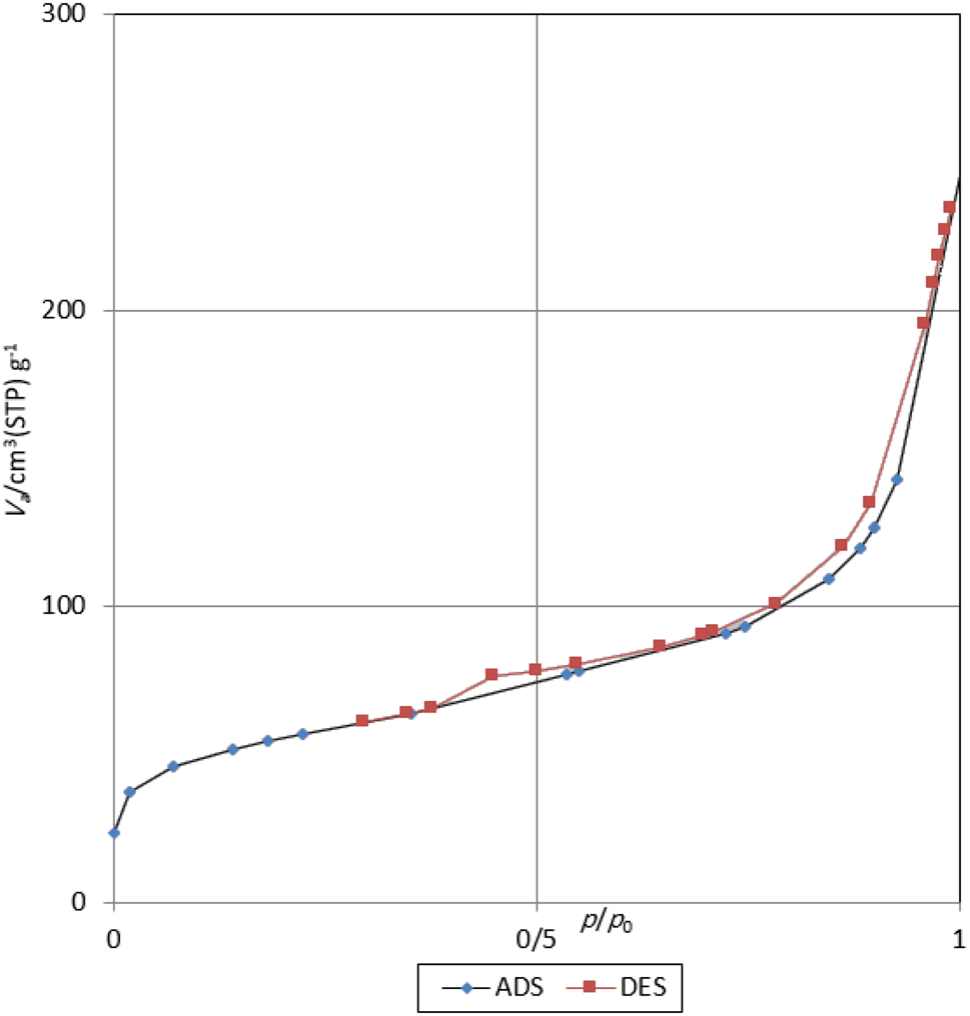
BET isotherm of the mesoporous Fe_3_O_4_@PMO-ICS-ZnO nanocatalyst (**1**).

Furthermore, the specific surface area and pore size of nano-ordered Fe_3_O_4_@PMO-ICS-ZnO material **1** were determined using N_2_ adsorption–desorption isotherms through Brunauer–Emmett–Teller (BET) and Barrett-Joyner-Halenda (BJH) methods (Fig. 7). In fact, the observed data demonstrated that this material has a typical mesoporous structure and a type IV isotherm, which is due to the presence of cylindrical pores on the mesoporous scale. Specific surface area, average pore size and total pore volume are approximately 194.9 m^2^ g^−1^, 7.3 nm, and 0.36 cm^3^ g^−1^, respectively. Comparison of the obtained BET and BJH data of Fe_3_O_4_@PMO-ICS-ZnO material **1** with the similar data for PMO-ICS^86^ and Fe_3_O_4_@PMO-ICS^87^ shows decreasment in the amount of surface area and total pore volume (Table 1). Therefore, it can be concluded that ZnO nanoparticles were firmly-embedded and fixed in the magnetic periodic mesoporous organosilica channels.

**Table 1:** Textural parameters of the PMO-ICS and Fe_3_O_4_@PMO-ICS-ZnO (**1**) samples.

Sample	BET surface area (m^2^/g)	Total pore vol. (cm^3^/g) (P/P_0_: 0.989)	Pore size (nm)
PMO-ICS	570.03	5.0	4.160
Fe_3_O_4_@PMO-ZnO	194.88	0.35	3.312

**Figure 8:**
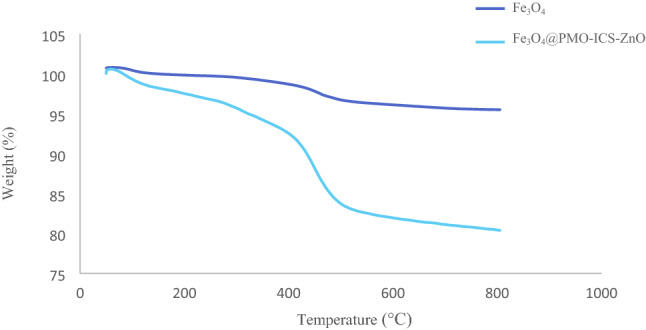
TGA curve of the Fe_3_O_4_@PMO-ICS-ZnO catalyst (**1**).

Thermogravimetric analysis (TGA) was measured for the prepared catalyst **1** at temperatures between 40 and 804 °C. Figure 8 shows three distinct weight loss for the Fe_3_O_4_@PMO-ICS-ZnO (**1**). The first step, with 3.56% weight loss between 40 and 270 °C, is corresponded to the removing of alcoholic or water solvents remaining from the extraction process. The second and main weight loss (14. 20%) at 270 to 570 °C region is attributed to the elimination of 1,3,5-tris(1,3-propylen) isocyanurate bridges incorporated into the PMO framework as well as condensation of its silanol groups. Finally, the last weight loss (2.26%) was observed in the range of 570–804 °C, which is attributed to more condensation of inorganic moieties present in the structure of catalyst **1** including silica and ZnO. These data clearly shows good thermal stability of the Fe_3_O_4_@PMO-ICS-ZnO, which is very important in design and application of recyclable heterogeneous catalytic systems.

**Figure 9:**
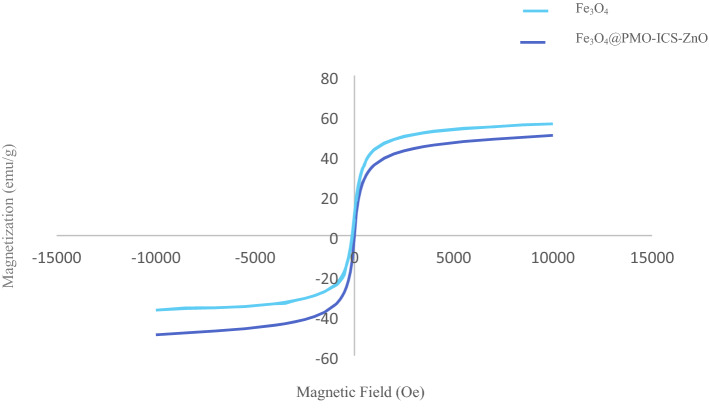
VSM magnetization curves for the Fe_3_O_4_ and Fe_3_O_4_@PMO-ICS-ZnO (**1**).

The magnetization of Fe_3_O_4_@PMO-ICS-ZnO (**1**) sample was measured using vibrating sample magnetometer (VSM) experiment. As can be seen in the Fig. 9, the hysteresis phenomenon was not observed. Instead, the observed “S” like curve at room temperature is also a proof of the paramagnetism of the prepared Fe_3_O_4_@PMO-ICS-ZnO. The saturation magnetization value was strongly enhanced by the external magnetic field strength at the low field region and found to be 49.56 emu/g for the Fe_3_O_4_@PMO-ICS-ZnO at − 10 to 10 KOe. Indeed, the magnetic property of the Fe_3_O_4_@PMO-ICS-ZnO is sufficiently enough to be easily separated by an external magnet from the reaction mixture.

### Optimization of conditions for the synthesis of tetrazole derivatives in the presence of magnetic Fe_3_O_4_@PMO-ICS-ZnO nanocatalyst (1)

After characterization of the magnetic Fe_3_O_4_@PMO-ICS-ZnO nanocatalyst (**1**), its efficiency was investigated for the synthesis of 1*H*-tetrazole derivatives. Therefore, the effect of different catalyst loadings, solvent and temperature on the yield and required time of reaction were systematically investigated in this step to find optimal conditions for the synthesis of 1*H*-tetrazole derivatives. Hence, the reaction of malononitrile (**2**, 1 mmol) and sodium azide (**3**, 1.2 mmol) 4-chlorobenzaldehyde (**4a**, 1 mmol), was selected as the model reaction. The results have been summarized in Table 2. Initially, the model reaction was examined in the absence of any catalyst under various conditions such as in EtOH at ambient temperature or under reflux conditions. The dependence of obtained yield of the model reaction to the catalyst and temperature was evident because of very low yields of the desired product, (*E*)-3-(4-chlorophenyl)-2-(1*H*-tetrazol-5-yl)acrylonitrile, **5a** under solvent-free conditions at room temperature or reflux conditions after prolonged reaction times (entries 1,2, Table 2). Interestingly, the obtained yield of desired product **5a** was significantly improved in the presence of Fe_3_O_4_@PMO-ICS-ZnO nanocatalyst (**1**) after very short reaction times (entries 3–5, Table 2). These findings demonstrate high catalytic activity of the magnetic nanocatalyst for the synthesis of the desired product **5a**, which originates from very good dispersion of active catalytic sites and proper surface area of the Fe_3_O_4_@PMO-ICS-ZnO. In the next experiments, the effect of different solvents such as water, EtOH, water/EtOH mixture, toluene, DMF, EtOAc and CH_3_CN as well as solvent-free conditions on the reaction rate was investigated (entries 6–12, Table 2). Indeed, both water and EtOH solvents afforded excellent yields of the desired product **5a** after same reaction time (entries 5, 6, Table 2). However, separation of the magnetic catalyst **1** is much easier in EtOH than water due to higher solubility of the desired 1*H*-tetrazole products at elevated temperatures and remaining the heterogeneous catalyst **1**. Therefore, 10 mg Fe_3_O_4_@PMO-ICS-ZnO loading in EtOH under reflux conditions was selected as the optimized conditions for further experiments (entries 3, 13, 14, Table 2). On the other hand, both pure PMO-ICS and magnetic PMO-ICS afforded lower yields of the desired product **5a** under similar conditions compared to the Fe_3_O_4_@PMO-ICS-ZnO nanocatalyst (**1**) (entries 15, 16, Table 2). All of these data show effective role of ZnO nanoparticles embedded in the thermally stable magnetic periodic mesoporous organosilica.

**Table 2:** Optimization of conditions in the reaction of malononitrile (**2**), NaN_3_ (**3**) and 4-chlorobenzaldehyde (**4a**) under different conditions.


Entry	Catalyst	Catalyst loading (mg)	Solvent	Temperature (°C)	Time	Yield^a^ (%)
1	–	–	Solvent-Free	rt	24 h	78
2	–	–	EtOH	Reflux conditions	20 h	62
**3**	**Fe**_**3**_**O**_**4**_**@PMO-ICS-ZnO**	**10**	**EtOH**	**Reflux conditions**	**3** min	**98**
4	Fe_3_O_4_@PMO-ICS-ZnO	15	EtOH	Reflux conditions	5 min	97
5	Fe_3_O_4_@PMO-ICS-ZnO	20	EtOH	Reflux conditions	5 min	98
6	Fe_3_O_4_@PMO-ICS-ZnO	10	H_2_O	Reflux conditions	5 min	95
7	Fe_3_O_4_@PMO-ICS-ZnO	10	H_2_O/EtOH (1:1)	Reflux conditions	40 min	80
8	Fe_3_O_4_@PMO-ICS-ZnO	10	Toluene	Reflux conditions	45 min	90
9	Fe_3_O_4_@PMO-ICS-ZnO	10	DMF	Reflux conditions	60 min	Trace
10	Fe_3_O_4_@PMO-ICS-ZnO	10	EtOAc	Reflux conditions	30 min	72
11	Fe_3_O_4_@PMO-ICS-ZnO	10	CH_3_CN	Reflux conditions	10 min	70
12	Fe_3_O_4_@PMO-ICS-ZnO	10	Solvent-Free	100	20 min	97
13	Fe_3_O_4_@PMO-ICS-ZnO	10	EtOH	60	7 min	86
14	Fe_3_O_4_@PMO-ICS-ZnO	10	EtOH	rt	10 min	97
15	PMO-ICS	10	EtOH	Reflux conditions	45 min	70
16	Fe_3_O_4_@PMO-ICS	10	EtOH	Reflux conditions	40 min	78

Reaction conditions: malononitrile (**2**, 1 mmol), NaN_3_ (**3**, 1.2 mmol) and 4-chlorobenzaldehyde (**4a**, 1 mmol) were added into the solvent (2.0 mL) in the presence of Fe_3_O_4_@ PMO-ICS-ZnO (**1**) unless otherwise stated.

^a^Isolated yields.

Significant values are in bold.

In the next step, the optimized conditions were expanded to other aromatic aldehydes **4b–o** to investigate the scope of reaction for the preparation of diverse 1*H*-tetrazole derivatives in the presence of magnetic Fe_3_O_4_@PMO-ICS-ZnO nanocatalyst (**1**). Excellent yields were obtained from a variety of aromatic carbocyclic or heterocyclic aldehydes **4a–o** under optimized conditions. As data in Table 3 show, high to excellent yields of the desired products **5a–o** were obtained within short reaction times. In this regard, aromatic aldehydes with carbocyclic ring bearing electron withdrawing groups **4a–g** and electron-deficient heterocycle **4h** were examined (entries 1–8, Table 3). On the other hand, aromatic aldehydes with carbocyclic ring bearing electron donating groups **4j–n** as well as electron-rich heterocycle **4o** survived to involve in the optimized conditions to afford the corresponding 1*H*-tetrazole derivatives **5j–o** (entries 10–15, Table 3).

**Table 3:** Synthesis of 5-substituted-1*H*-tetrazole derivatives **5a–o** catalyzed by the Fe_3_O_4_@PMO-ICS-ZnO nanocatalyst (**1**) via the three components reaction strategy.

Entry	Aldehyde 4 (Ar)	Product 5	Time (min)	Yield^a^ (%)	MP °C (Obs.)	MP °C (Lit.)
1	4-ClC_6_H_4_ **4a**		5	98	157–159	158–160^110^
2	4-FC_6_H_4_ **4b**		5	78	175–177	176–179^111^
3	3-BrC_6_H_4_ **4c**		5	80	162–163	165–167^112^
4	2-ClC_6_H_4_ **4d**		15	97	171–173	175–177^64^
5	2,4-Cl_2_C_6_H_3_ **4e**	**5e**	30	95	150–152	142–143^113^
6	4-NO_2_C_6_H_4_ **4f**		25	95	167–169	166–168^112^
7	3-NO_2_C_6_H_4_ **4g**		30	92	161–163	159–163 ^66^
8			8	88	187–188	185–186^113^
9	C_6_H_5_ **4i**	**5i**	5	97	165–167	168–170^112^
10	4-CH_3_C_6_H_4_ **4j**		5	97	186–188	189–191^114^
11	4-MeOC_6_H_4_ **4k**		20	90	150–152	153–155^66^
12	4-HOC_6_H_4_ **4l**		5	88	161–164	159–161^112^
13	2-MeOC_6_H_4_ **4m**		7	90	157–159	150–152^115^
14	4-Me_2_NC_6_H_4_ **4n**		25	88	167–168	171–172^113^
15			15	80	88–90	85–86^113^

Reaction conditions: malononitrile (**2**, 1 mmol), aldehyde (**4**, 1 mmol), and NaN_3_ (**3**, 1.2 mmol) were added into the EtOH (2.0 mL) in the presence of 10 mg of Fe_3_O_4_@ PMO-ZnO (**1**).

^a^Isolated yields.

### Proposed mechanism for the preparation of 5-substituted-1H-tetrazole derivatives catalyzed by Fe_3_O_4_@PMO–ICS–ZnO (1)

**Figure 10:**
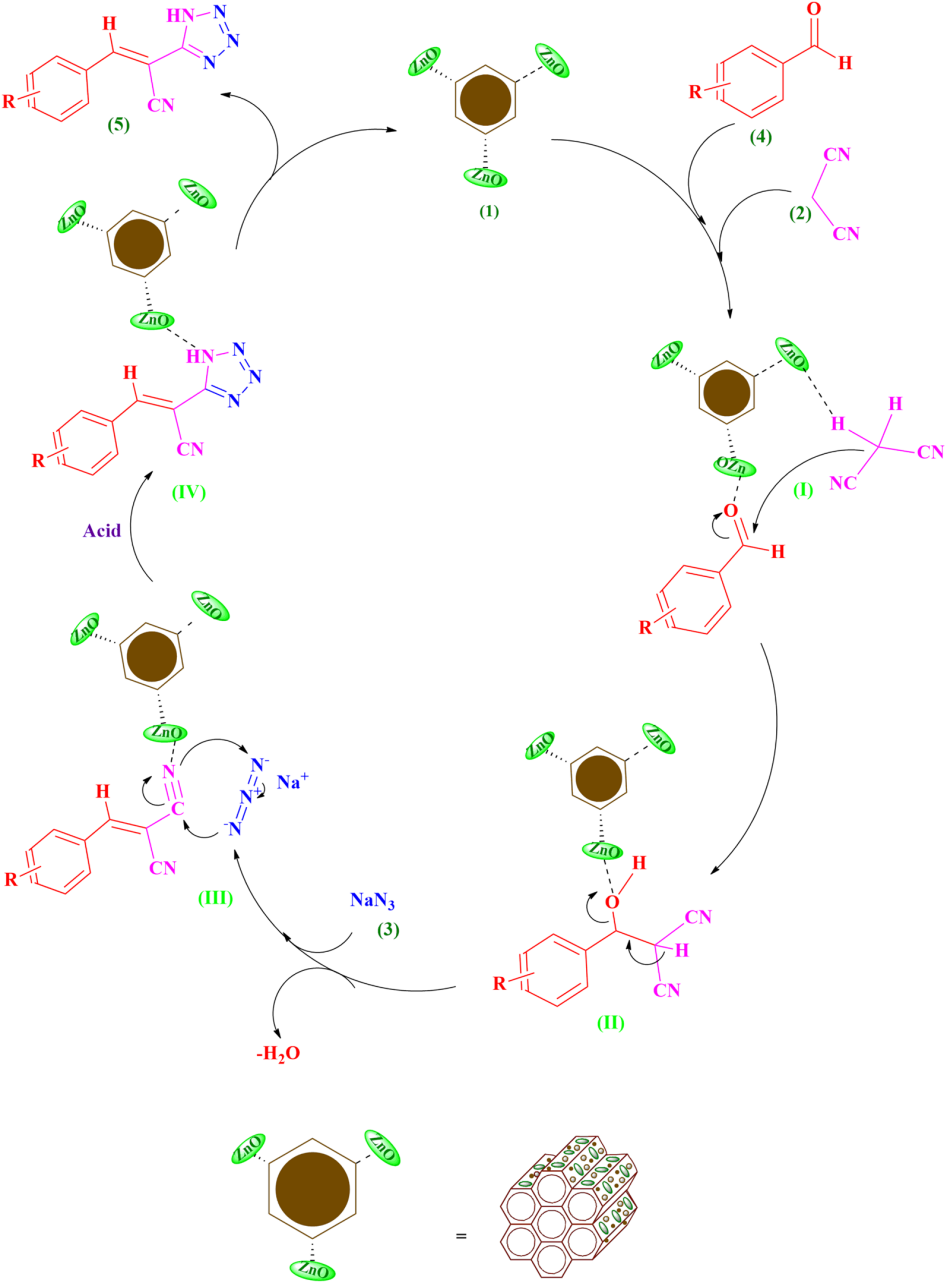
A reasonable mechanism for the one-pot preparation of (*E*)-2-(1*H*-tetrazole-5-yl)-3-aryl/heteroarylacrylenenitrile derivatives **5** using the magnetic nano-ordered Fe_3_O_4_@PMO–ICS–ZnO catalyst (**1**).

A trajectory for the cascade one-pot preparation of (*E*)-2-(1*H*-tetrazole-5-yl)-3-aryl/heteroarylacrylenenitrile derivatives **5a–o** using Lewis acidic ZnO nanoparticles embedded in the magnetic isocyanurate-based periodic mesoporous organosilica (Fe_3_O_4_@PMO–ICS–ZnO, **1**) is shown in Fig. 10. In the first reaction step, the aromatic aldehydes (**4**) are activated by the Fe_3_O_4_@PMO–ICS–ZnO step by step to condensate with the malononitrile C–H acid (**2**) and finally affording the Knoevenagel aryl/heteroarylidine malononitrile intermediate (**III**). This intermediate is subsequently involved in the concerted [3 + 2] cycloaddition with sodium azide (**3**) to produce 5-membered tetrazole ring.

### Investigating of the reusability of magnetic Fe_3_O_4_@PMO–ICS–ZnO (1) for the synthesis of 5-substituted-1***H***-tetrazole derivatives 5

**Figure 11:**
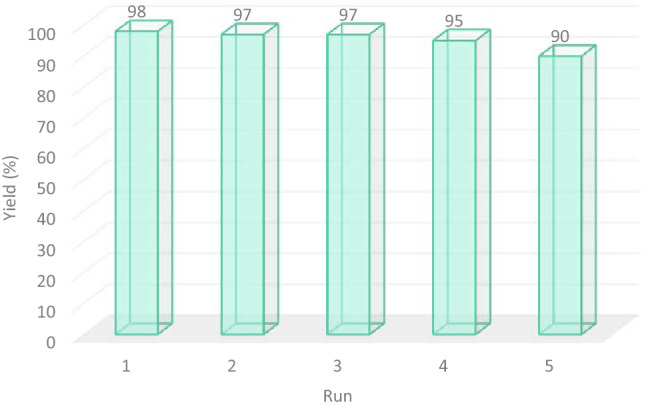
Reusability data for the magnetic Fe_3_O_4_@PMO-ICS-ZnO mesoporous catalyst (**1**) in the synthesis of **5a**.

As a part of our study, the reusability of magnetic Fe_3_O_4_@PMO–ICS–ZnO (**1**) for the synthesis of (*E*)-3-(4-chlorophenyl)-2-(1*H*-tetrazol-5-yl)acrylonitrile **5a** was investigated in the next step. The catalyst **1** was easily separated after completion of the model reaction under optimized conditions by an external magnet. The recycled Fe_3_O_4_@PMO–ICS–ZnO was separately dispersed in EtOH and EtOAc for 15 min, respectively. The recycled catalyst was kept in an oven at 60 °C for 1 h and then reused for the next runs. The results of the catalyst recycling for five consecutive runs are given in Fig. 11. As data in Fig. 10 demonstrate, only a slight decrease in reaction yield was observed after fourth run. These findings indicate the structural stability of magnetic the Fe_3_O_4_@PMO–ICS–ZnO (**1**) during synthesis of 5-substituted-1*H*-tetrazole derivatives **5**.

### Comparison of the catalytic activity of nano-ordered Fe_3_O_4_@PMO–ICS–ZnO (1) in the synthesis of tetrazole derivatives with other catalytic systems

Table 4 compares the previously reported methods for the synthesis of tetrazole **5a** with the present protocol. It is apparent that high to excellent yields, avoiding the use of toxic or carcinogenic solvents such as DMF, short reaction time and easy separation of the catalyst from the reaction mixture are the advantages of the present protocol compared to the most of previously reported methods.

**Table 4:** Comparison of the catalytic efficiency of the Fe_3_O_4_@PMO-ZnO (**1**) with other heterogeneous or homogeneous catalytic systems for the synthesis of **5a**.

Entry	Catalyst	Catalyst loading (mg)	Conditions	Time (h)	Yield 4a (%)	Ref.
1	NiO nanoparticles	4.5	DMF/70 °C	6	87	^65^
2	Silica molybdic acid	200	H_2_O/MW/50 °C	20 min	91	^66^
3	Zn (Metallic)	130.7	H_2_O/50 °C	3	68	^110^
4	Fe(OAc)_2_	17.4	DMF/H_2_O (9:1) 80 °C	24	89	^116^
5	(CuOTf)_2_.C_6_H_6_	36.2	Toluene/rt	7	81	^117^
6	Montmorillonite K 10	10.0	H_2_O	17	29	^118^
			DMF		38	
7	Cu(OAc)_2_	45.4	DMF/120 °C	12	96	^119^
8	Catalyst-Free	_	Solvent-Free	24 h	78	_
9	OPNSA	45.4	Solvent-Free	8	90	^120^
10	H_3_PW_12_O_40_	20.2	Solvent-Free/120 °C	6	93	^121^
11	NH–Cu(II)@MNP	20.0	EtOH/80 °C	5	92	^111^
12	**Fe**_**3**_**O**_**4**_**@PMO–ICS–ZnO**	**10.0**	**EtOH/reflux**	**3**	**98**	**This work**

Significant values are in bold.

## Experimental

### Reagents and instruments

All chemical substances and reagents with high purity were purchased from Merck or Aldrich and used as received, except for liquid aldehydes which were distilled before their using. The progress of reactions and the purity of the obtained products were monitored by thin layer chromatography (TLC) using Merck aluminum plates coated with 0.2 mm silica gel F254. Melting points were measured using an Electrothermal 9100 device and are uncorrected. Characterization of the magnetic catalyst **1** as well as identification of products was performed using KBr discs on a Shimaduzu FTIR-8400S spectrometer. A Bruker DRX-500 Avance spectrometer was used for recording of ^1^H NMR (500 MHz) and ^13^C NMR (125 MHz) spectra of products in DMSO-*d*_6_ at ambient temperature. The BET specific surface area analysis was performed using ASAP 2020™ instrument. Thermal gravimetric analysis data was obtained by a Bahr company STA 504 equipment. X-Ray diffraction pattern was prepared using a STOE apparatus with CuKα radiation source. Field emission scanning electron microscopy images were recorded by a Zeiss (EM10C) device. VSM analysis was performed using a Lakeshore 7410 series instrument (Supplementary file).

### Typical procedure for the preparation of PMO-ICS

The periodic mesoporous organosilica denoted PMO-ICS was prepared according to the method introduced by Jaroniec^122^. This PMO-ICS was synthesized by self-assembly of tris[3-(trimethoxysilyl)propyl] isocyanurate (ICS, Aldrich), tetraethyl orthosilicate (TEOS, Aldrich) in the presence of poly(ethylene oxide)-poly(propylene oxide)-poly(ethylene oxide) triblock copolymer (Pluronic 123, Aldrich, average Mw ≅ 5800 Dalton) under acidic conditions. In a typical experiment, P123 (2.0 g) was added into a 250 mL beaker and a mixture of deionized water (15 mL) and hydrochloric acid solution (2.0 M, 60 mL) was slowly added and stirred until P123 is completely dissolved. Then, ICS (0.01 mol, 3.08 g) and TEOS (0.03 mol, 3.12 g) were simultaneously added dropwise into the obtained solution. After that, the obtained white gel and precipitates was stirred at room temperature for 24 h in a round bottom flask. Then, the mixture was aged at 100 °C for 48 h without stirring. The solid was filtered off and washed thoroughly with hot EtOH/HCl (60 mL of 96% EtOH and 2 mL of 12.0 M HCl) using a soxhelet apparatus for 72 h to remove the surfactant molecules. The obtained white powder was finally dried in air at 100 °C overnight.

### General procedure for the preparation of magnetic Fe_3_O_4_@PMO-ICS

PMO-ICS (2.0 g) was dispersed in toluene (20 mL) at room temperature. After 15 min stirring, FeCl_2_.4H_2_O (2.0 g) and FeCl_3_.6H_2_O (4.0 g) were added to the above mixture under nitrogen atmosphere. The reaction mixture was heated in an oil bath at 80 °C during stirring, Then, ammonia solution (25% w/w, 20 mL) was added dropwise into the mixture over a period of 30 min until pH 11.0 is reached and allowed to stir for one hour at the same temperature. The obtained black precipitate was finally collected by an external magnet, washed with deinozed water and EtOH and then dried at 100 °C for 2 h.

### Preparation of ZnO nanoparticles embedded in the mesoporous Fe_3_O_4_@PMO-ICS (Fe_3_O_4_@PMO–ICS–ZnO, 1)

At this stage, Zn(OAc)_2_ in the presence of PEG-600 surfactant was used to embed ZnO nanoparticles into the channels of Fe_3_O_4_@PMO-ICS. Zn(OAc)_2_ (0.1 g) and PEG (0.1 g) were added to a mixture of Fe_3_O_4_@PMO-ICS (3.0 g) dispersed in twice-distilled water (50 mL). Then, NH_3_ solution (25% w/w) was added dropwise to the obtained mixture and adjusting pH to 10.0 and then heated for 8 h at 80 °C. Finally, the mixture was cooled to ambient temperature and the obtained crimson Fe_3_O_4_@PMO–ICS–ZnO powder was magnetically separated. The powder was washed with deionized water twice and then dried at 100 °C for 2 h.

### General procedure for the synthesis of 5-substituted-1***H***-tetrazoles derivatives 5a-o catalyzed by Fe_3_O_4_@PMO–ICS–ZnO (1)

In a single-neck round-bottom 10 mL flask equipped with a condenser, a mixture of malononitrile (**2**, 1 mmol), sodium azide (**3**, 1.2 mmol) and aldehyde (**4**, 1 mmol) was heated in the presence of Fe_3_O_4_@PMO–ICS–ZnO catalyst **1** (10 mg) in EtOH under reflux conditions for the time indicated in Table 3. The reaction progress was monitored by TLC (Eluent: EtOAc/n-hexane, 1:3). After completion of the reaction, Fe_3_O_4_@PMO–ICS–ZnO catalyst was easily separated from the reaction mixture using an external magnet and the desired product **5** was crystallized by dropwise adding of distilled water to the ethanolic solution. The structure of products **5a–o** was confirmed by melting point as well as FTIR, ^1^H NMR and ^13^C NMR spectroscopy.

## Conclusions

Novel magnetic Fe_3_O_4_@PMO–ICS–ZnO nano-ordered catalyst was prepared through a modified environmentally-benign procedure and properly characterized. The catalytic activity of the new thermally stable Fe_3_O_4_@PMO–ICS–ZnO nanomaterial was demonstrated in the one-pot synthesis of tetrazole derivatives through cascade condensation and concerted 1,3-cycloaddition reactions as a representative of the Click Chemistry concept. Different aromatic aldehydes survived to involve smoothly in the optimized conditions affording the corresponding 5-substituted-1*H*-tetrazole derivatives in high to quantitative yields and good purity. Low catalyst loading, the use of green solvents such as EtOH or water instead of carcinogenic DMF and short reaction time as well as easy separation and recyclability of the catalyst for at least five consecutive runs without significant loss of its activity are notable advantages of this new protocol compared to other recent introduced procedures.

## Supplementary Information

Supplementary Information.
