# Supplementary material for: Synthesis of (E)-2-(1H-tetrazole-5-yl)-3-phenylacrylenenitrile derivatives catalyzed by new ZnO nanoparticles embedded in a thermally stable magnetic periodic mesoporous organosilica under green conditions
Source: Sci Rep. 2022 Jun 24;12:10723. doi: 10.1038/s41598-022-13011-9 (PMC9232489; doi:10.1038/s41598-022-13011-9)
Supplement: Supplementary file 1 — Supplementary Information. [file 41598_2022_13011_MOESM1_ESM.docx]

***Electronic Supporting Information***

**Synthesis of (*E*)-2-(1*H*-tetrazole-5-yl)-3-phenylacrylenenitrile derivatives catalyzed by new ZnO nanoparticles embedded in a thermally stable magnetic periodic mesoporous organosilica under green conditions**

Sajedeh Safapoor, Mohammad G. Dekamin, ^*^ Arezoo Akbari and M. Reza Naimi-Jamal

*Pharmaceutical and Heterocyclic Compounds Research Laboratory, Department of Chemistry, Iran University of Science and Technology, Tehran 16846-13114, Iran*

*^*^ E-mail:* [*mdekamin@iust.ac.ir*](mailto:mdekamin@iust.ac.ir)

**Contents**

[General data 2](#_Toc72445647)

Characterization of magnetic ZnO nanoparticles embedded in periodic mesoporous orgnosilica...............................................................................................................................................…...3

[General procedure for the synthesis of 5-substituted-1*H*-tetrazoles derivatives **5a-o** catalyzed by Fe_3_O_4_@PMO–ICS–ZnO (**1**)………………………………………………………………………………..…………………………………..7](#_Toc72445648)

Characterization of the selected compounds…..……………………………………………….....………………………………….……….8

General data

**Reagents and instruments**

All chemical substances and reagents with high purity were purchased from Merck or Aldrich and used as received, except for liquid aldehydes which were distilled before their using. The progress of reactions and the purity of the obtained products were monitored by thin layer chromatography (TLC) using Merck aluminum plates coated with 0.2 mm silica gel F254. Melting points were measured using an electro thermal 9100 device and are uncorrected. Characterization of the magnetic catalyst **1** as well as identification of products was performed using KBr discs on a Shimaduzu FTIR-8400S spectrometer. A Bruker DRX-500 Avance spectrometer was used for recording of ^1^H NMR (500 MHz) and ^13^C NMR (125 MHz) spectra of products in DMSO-*d_6_* at ambient temperature. The BET specific surface area analysis was performed using ASAP 2020^TM^ instrument. Thermal gravimetric analysis data was obtained by a Bahr company STA 504 equipment. X-Ray diffraction pattern was performed using a STOE apparatus with CuKα radiation source. Field emission scanning electron microscopy images were recorded by a Zeiss (EM10C) device. VSM analysis was performed using a Lakeshore 7410 series instrument.

**Typical Procedure for the Preparation of PMO-ICS**

The periodic mesoporous organosilica denoted PMO-ICS was prepared according to the method introduced by Jaroniec^123^. This PMO-ICS was synthesized by self-assembly of tris[3-(trimethoxysilyl)propyl] isocyanurate (ICS, Aldrich), tetraethyl orthosilicate (TEOS, Aldrich) and the poly(ethylene oxide)-poly(propylene oxide)-poly(ethylene oxide) triblock copolymer (Pluronic 123, Aldrich, average Mw ≅ 5800 Dalton) under acidic conditions. In a typical experiment, P123 (2.0 g) was added into a 250 mL beaker and a mixture of deionized water (15 mL) and hydrochloric acid solution (2.0 M, 60 mL) was slowly added and stirred until P123 is completely dissolved. Then, ICS (0.01 mol, 3.08 g) and TEOS (0.03 mol, 3.12 g) were simultaneously added dropwise into the obtained solution. After that, the obtained white gel and precipitates was stirred at room temperature for 24 h in a round bottom flask. Then, the mixture was aged at 100 °C for 48 h without stirring. The solid was filtered off and washed thoroughly with hot EtOH/HCl (60 mL of 96% EtOH and 2 mL of 12.0 M HCl) using a soxhelet apparatus for 72 h to remove the surfactant molecules. The obtained white powder was finally dried in air at 100 ^o^C overnight.

**General procedure for the preparation of magnetic Fe_3_O_4_@PMO-ICS**

PMO-ICS (2.0 g) was dispersed in toluene (20 mL) at room temperature. After 15 minutes stirring, FeCl_2_.4H_2_O (2.0 g) and FeCl_3_.6H_2_O (4.0 g) were added to the above mixture under nitrogen atmosphere. The reaction mixture was heated in an oil bath at 80 °C during stirring, Then, ammonia solution (25% w/w, 20 mL) was added dropwise into the mixture over a period of 30 minutes until the pH reached 11.0 and allowed to stir for one hour at the same temperature. The obtained black precipitate was finally washed with deinozed water and EtOH and then dried at 100 °C for 2 h.

**Preparation of ZnO nanoparticles embedded in mesoporous Fe_3_O_4_@PMO-ICS (Fe_3_O_4_@PMO–ICS–ZnO, 1)**

At this stage, Zn(OAc)_2_ in the presence of PEG-600 surfactant was used to embed ZnO nanoparticles into the channels of Fe3O4@PMO-ICS. Zn(OAc)_2_ (0.1 g) and PEG (0.1 g) was added to a mixture of Fe_3_O_4_@PMO-ICS (3.0 g) dispersed in twice-distilled water (50 mL). Then, NH_3_ solution (25% w/w) was added dropwise to the obtained mixture and adjusting pH = 10.0 and then heated for 8 h at 80 °C. Finally, the mixture was cooled to ambient temperature and the obtained crimson Fe_3_O_4_@PMO–ICS–ZnO powder was magnetically separated. The powder was washed with deionized water twice and then dried at 100 °C for 2 h.


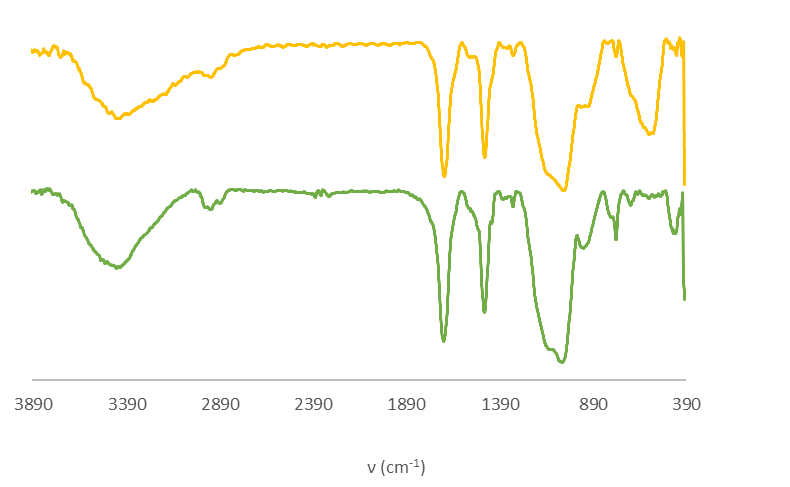


PMO-ICS

Fe_3_O_4_@PMO-ICS-ZnO

Transmittance (%)

**Figure 1.** FTIR spectra of the PMO-ICS and magnetic Fe_3_O_4_@PMO-ICS-ZnO nanoporous catalyst (**1**).

| Element | W (%) |
| --- | --- |
| C | 18.90 |
| N | 8.51 |
| O | 50.43 |
| Si | 9.67 |
| Fe | 11.78 |
| Zn | 1.7 |


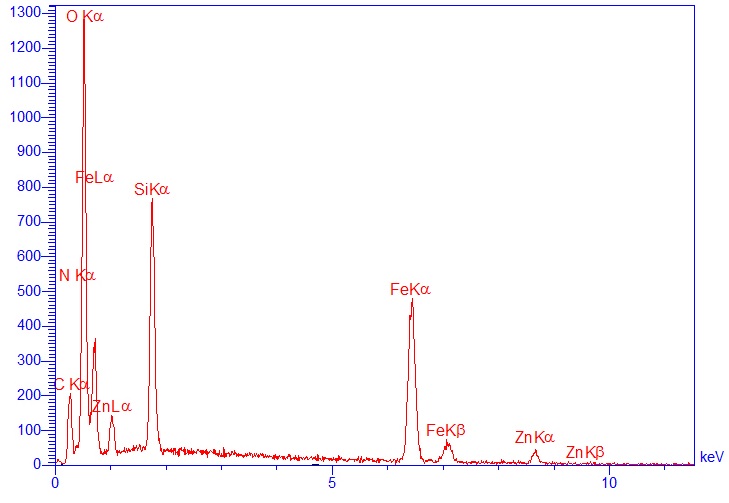


CPS (a.u.)

**Figure 2.** EDX spectrum of the magnetic Fe_3_O_4_@PMO-ICS-ZnO mesoporous catalyst (**1**).


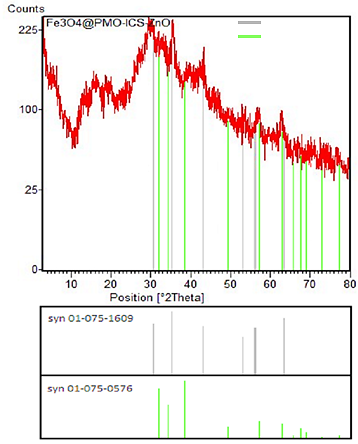


Fe_3_O_4_

ZnO

_o_2Theta

**Figure 3.** X-ray powder diffraction (XRD) pattern for Fe_3_O_4_@PMO-ICS-ZnO nanoporous catalyst (**1**).


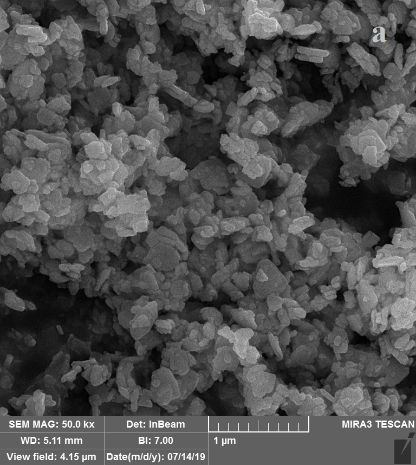

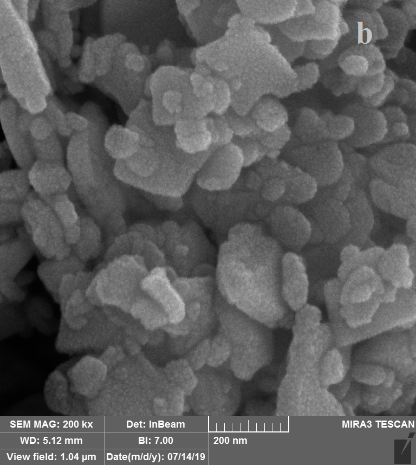

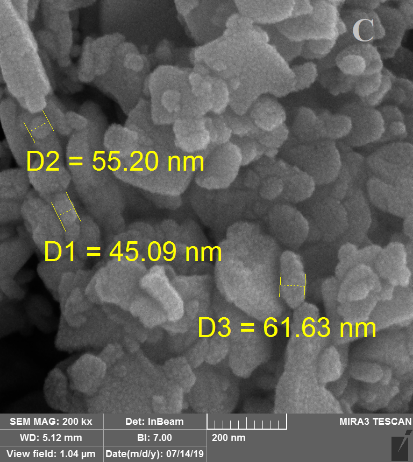


**Figure 4.** FESEM images of magnetic nanoporous Fe_3_O_4_@PMO-ICS-ZnO catalyst (**1**).


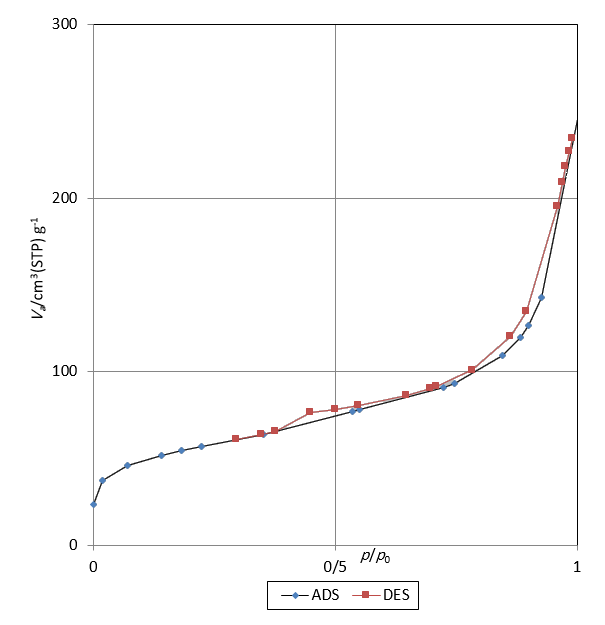
**Figure 5.** BET isotherm of the mesoporous Fe_3_O_4_@PMO-ICS-ZnO nanocatalyst (**1**).

Textural parameters of the PMO-ICS and Fe_3_O_4_@PMO-ICS-ZnO (**1**) samples

| Sample | BET surface area (m^2^/g) | Total pore vol. (cm^3^/g)  (P/P_0_: 0.989) | Pore size (nm) |
| --- | --- | --- | --- |
| PMO-ICS | 570.03 | 5.0 | 4.160 |
| Fe_3_O_4_@PMO-ZnO | 194.88 | 0.35 | 3.312 |

Weight (%)


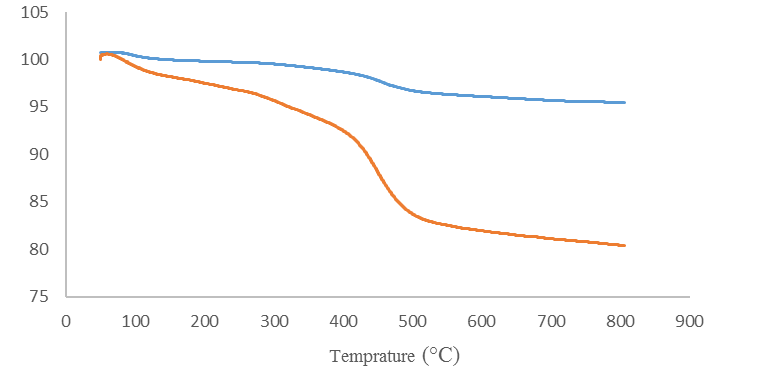


Fe_3_O_4_

Fe_3_O_4_@PMO-ICS-ZnO

**Figure 6**. TGA curve of Fe_3_O_4_@PMO-ICS-ZnO catalyst (**1**).

Fe_3_O_4_

Fe_3_O_4_@PMO-ICS-ZnO


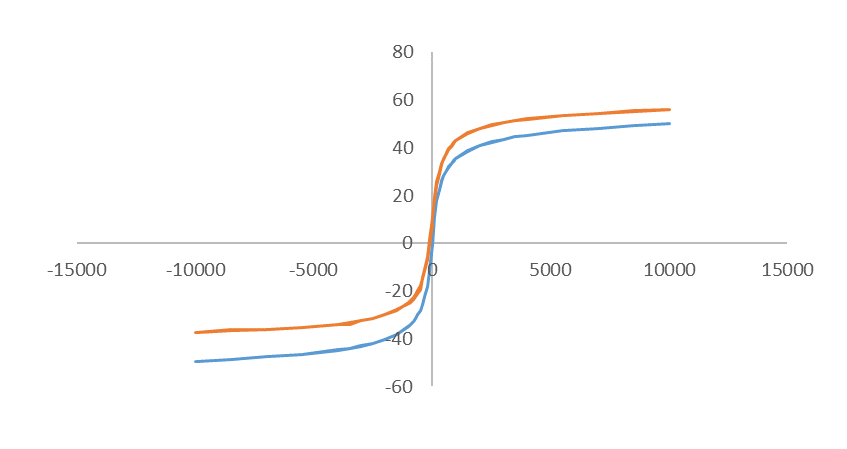


Magnetization (emu/g)

Magnetic Field (Oe)

**Figure 7.** VSM magnetization curves for the Fe_3_O_4_ and Fe_3_O_4_@PMO-ICS-ZnO (**1**).

**General procedure for the synthesis of 5-substituted-1*H*-tetrazoles derivatives 5a-o catalyzed by Fe_3_O_4_@PMO–ICS–ZnO (1)**

In a single-neck round-bottom 10 mL flask equipped with a condenser, a mixture of malononitrile (**2**, 1 mmol), sodium azide (**3**, 1.2 mmol) and aldehyde (**4**, 1 mmol) was heated in the presence of Fe_3_O_4_@PMO–ICS–ZnO catalyst **1** (10 mg) in EtOH under reflux conditions for the time indicated in **Table 3**. The reaction progress was monitored by TLC (Eluent: EtOAc/n-hexane, 1:3). After completion of the reaction, Fe_3_O_4_@PMO–ICS–ZnO catalyst was easily separated from the reaction mixture using an external magnet and the desired product **5** was crystallized by dropwise adding of distilled water to the ethanolic solution. The structure of products **5a-o** was confirmed by melting point as well as FTIR, ^1^H NMR and ^13^C NMR spectroscopy.

### **Characterization of the selected compounds**

**(*E*)-3‑Phenyl‑2(1*H*‑tetrazole‑5‑yl) acrylonitrile** **(5a)**

Cream solid powder; **Mp:** 165-167 °C; **FT-IR (KBR) ν:** 3283, 3027, 2206, 1592, 1473, 984, 826, 676; **^1^H NMR** **(500 MHz, DMSO-*d_6_*):** δ (ppm) 3.44 (s, br, NH overlap with solvent), 7.91-7.96 (d, *J* = 7.4 Hz, 3H), 7.43-7.55 (m, 2H), 8.10 (d, *J* = 12.3 Hz, 1H).

**(*E*)-3-(4-Chlorophenyl)-2-(1*H*-tetrazole-5-yl) acrylonitrile (5b)**

Pale yellow powder; **Mp:** 158-160 °C; **FT-IR (KBR) ν:** 3158, 2267, 1584,1426,1123,930,810,691, 652. **^1^H NMR** **(500 MHz, DMSO-*d_6_*):** δ (ppm) 3.50 (s, br, NH overlap with solvent), 7.72 (d, *J* = 8.5 Hz, 2H), 7.95 (d, *J* = 8.5 Hz, 2H), 8.56 (s,1H, CH).

**(*E*)-3-(4-methylphenyl‑2‑(1*H*‑tetrazole‑5‑yl) acrylonitrile** **(5e)**

Cream solid powder; **Mp:** 189-191 °C; **FT-IR (KBR) ν:** 3033, 2219,1585,1556,1376,1218,1184,813,609; **^1^H NMR** **(500 MHz, DMSO-*d_6_*):** δ (ppm) 2.44(s, 3H, CH_3_), 3.31 (s, br, NH overlap with solvent), 7.44 (d, *J* = 8.1 Hz, 2H), 7.86 (d, *J* = 8.1 Hz, 2H), 8.47(s, 1H, CH).


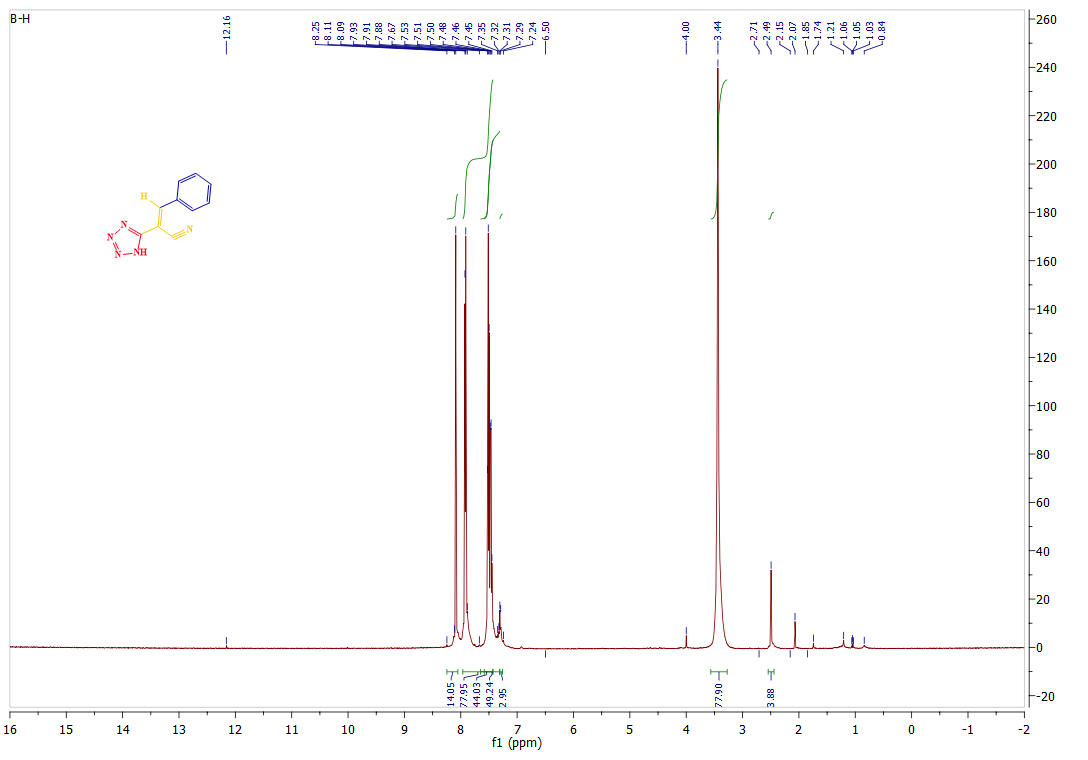


**^1^H NMR spectra of compound 5a.**

^
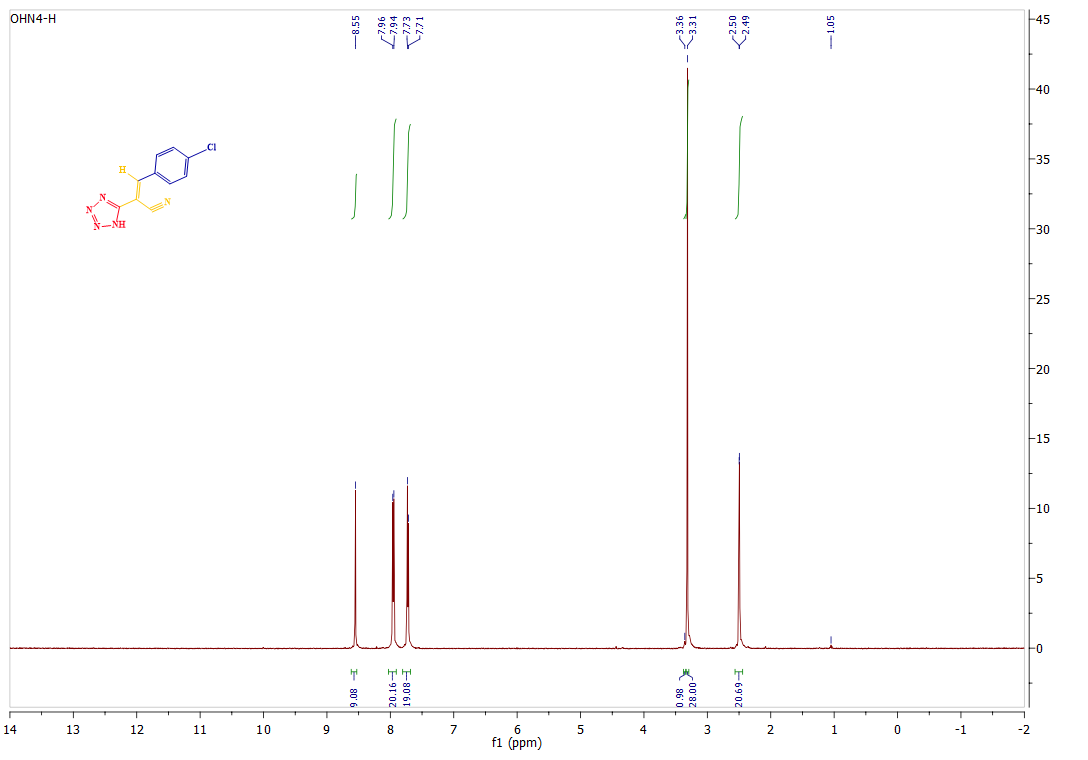

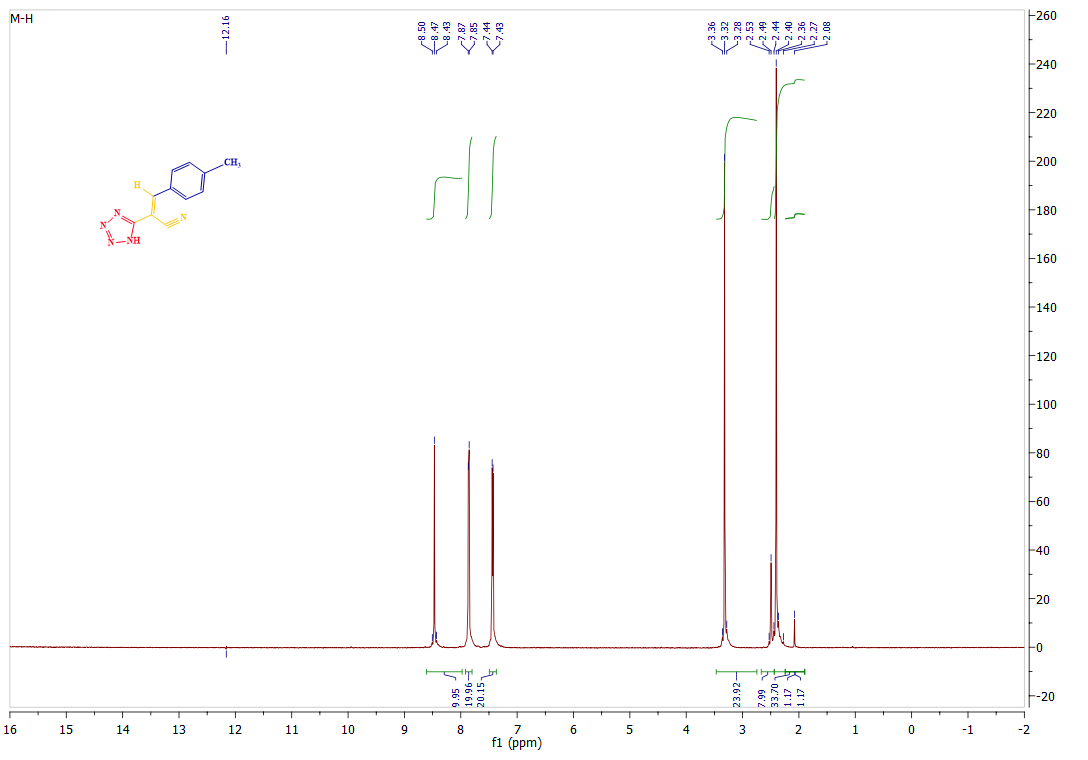
^**^1^H NMR spectra of compound 5b.**

**^1^H NMR spectra of compound 5e.**
